# Oxo-Hydrazyl as a Substitute for the Stable Free Radicals Employed in Measuring Total Antioxidant Activity

**DOI:** 10.3390/antiox14040463

**Published:** 2025-04-12

**Authors:** Petre Ionita

**Affiliations:** Faculty of Chemistry, University of Bucharest, Panduri 90, 050663 Bucharest, Romania; petre.ionita@chimie.unibuc.ro

**Keywords:** DPPH, ABTS, betaine, zwitterionic, TAC, radical, antioxidant

## Abstract

Numerous standardized methods for evaluating antioxidant capacity are available, some of the most used methods in this regard employ stable free radicals, like 2,2-diphenyl-1-picrilhydrazyl free radical (DPPH·) or 2,2′-azino-bis(3-ethylbenzothiazoline-6-sulfonic acid cation radical (ABTS·^+^). However, some challenges can arise in taking correct and reproducible measurements due to the well-known unspecific reactivity of these free radicals. In pursuit of improving and expanding such methods, in this work is proposed a highly intense colored zwitterionic derivative of the DPPH· free radical, as a replacement for DPPH· and ABTS·^+^ derivatives. A discussion and comparison of the recognized methods are presented, demonstrating the very good potential of this non-radical compound.

## 1. Introduction

**Figure 1:**
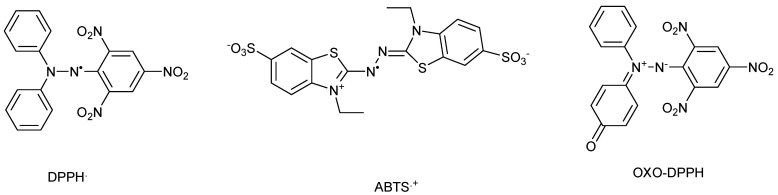
Chemical structure of DPPH·, ABTS·^+^, and oxo-DPPH.

Highly intense colored compounds, like 2,2-diphenyl-1-picrilhydrazyl free radical (DPPH·) or 2,2′-azino-bis(3-ethylbenzothiazoline-6-sulfonic acid cation radical (ABTS·^+^) are the most common stable free radicals used in evaluating of the total antioxidant capacity (TAC) of a large variety of pure, natural, or artificial compounds (like vitamins, polyphenols, etc.), complex mixtures (like plant extracts, wines, food, etc.) [1,2,3], or even materials [4,5,6] (Figure 1).

These TAC methods are based on the fading color intensity of the above radicals in interactions with compounds or materials that are prone to oxidative processes [7]. Standardized protocols are available and different studies regarding their limits have been published [2,3].

An ABTS·^+^ radical cation is a water-soluble free radical with several absorption bands (peaks) in the visible domain, usually at 414, 645, 738, and 811 nm, while a DPPH· free radical is soluble in organic solvents and has a single absorption maximum around 516 nm (though small differences can be present due to the solvent used). Depending on concentration, an aqueous solution of ABTS·^+^ radical cation is perceived as green-blue, while an organic solution of the DPPH· free radical is pink-violet.

For natural systems, oxidative stress is regarded as an imbalance between reactive oxygen or nitrogen species and the defense antioxidant systems. For this reason, evaluating the antioxidant properties of a specific system is important in preventing oxidative stress and assessing the potential to confer protective benefits against possible problems that can arise, including those associated with age-related diseases [3].

Multiple oxidant sources are present in nature, mainly due to the atmospheric oxygen, leading to the generation of free radicals, like hydroxyl, peroxyl, nitric oxides, etc. In biological systems, Pryor et al. [2] identified four general sources of antioxidants, the most common of which are enzymes, other large molecules like proteins, small molecules (vitamins and polyphenols), and hormones.

This is regarded as a general knowledge and it is widely accepted that antioxidants from foods appear to be quite useful in preventing several pathologies and aging-related disorders and neurological diseases. Antioxidants significantly delay or prevent substrate oxidation when they are present at low concentrations [8].

**Figure 2:**
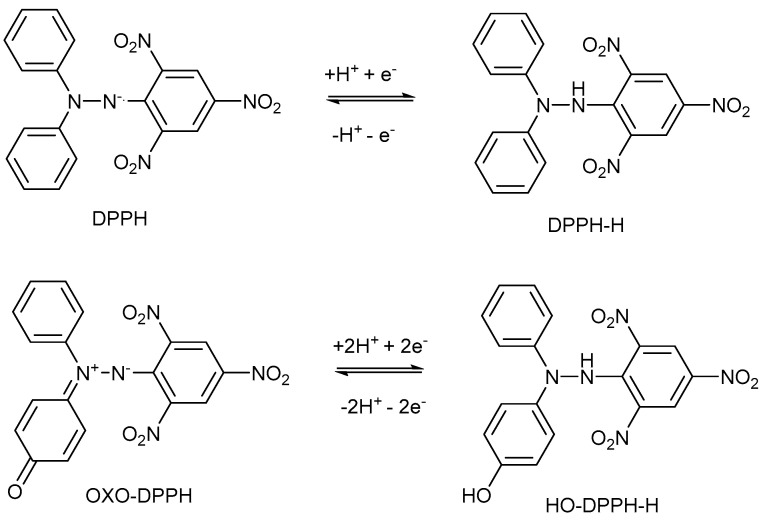
Reversible redox processes of DPPH· and oxo-DPPH.

Oxo-DPPH is a stable non-radical organic compound (Figure 1); it is highly intensely colored and can be involved in redox processes, similarly to DPPH·, as shown in Figure 2. However, there is a difference in terms of the numbers of electron involved in such processes—for DPPH· or ABTS·^+^ free radicals, a single electron is used, while for oxo-DPPH, two electrons are required.

In this work, the oxo-DPPH derivative is proposed as a new non-radical organic compound that can be used in TAC measurements (Figure 1). The benefits and drawbacks of using this compound are discussed, showing that there are certain advantages to replacing well-known DPPH· or ABTS·^+^ free radicals with the oxo-DPPH derivative.

## 2. Results and Discussion

### 2.1. General Characteristics of DPPH·, ABTS·^+^, and Oxo-DPPH Derivative, and Their Mechanism of Action

Antioxidants function by transforming the existing free short-lived radicals (such as those generated in living cells) into less damaging molecules, thereby inhibiting the generation of additional free radical species and blocking the radical reaction chain [2,3,9]. Data from the literature present interesting insights about the mechanism of radical scavenging activities, as found using complementary methods like nuclear magnetic resonance (NMR), UV–Vis, cyclic voltammetry, and electron spin resonance (ESR) [10,11,12,13,14,15]. Using NMR [10], it was found that the order of radical-scavenging ability was ascorbic acid > R-tocopherol > ethyl gallate > catechin (i.e., for tea leaves and using DPPH· assay). By ESR, it was found that no correlation can be found between the results obtained by DPPH· and ABTS·^+^ assays. It is worth to remind that these assays are practically used in single electron transfer reactions [11].

Other comparative studies are available, demonstrating that, in the case of phenolic compounds, their antioxidant activity closely depends on their chemical and stereochemical structures [16]. In addition, in a comparative study using many different approaches (trolox equivalent antioxidant capacity (TEAC), DPPH·, oxygen radical absorbance capacity (ORAC), resistance to hemolysis, and other assays), it was found that in the case of DPPH· assay the results were almost always lower than the others [17]. In a similar way, cyclic voltammetry might provide useful information [18], showing the number of electrons transferred in such reactions.

The redox potential of the ABTS·^+^ is around 0.5 V [19,20], while for the DPPH· free radical it is about 0.34 V [21]; a similar value of 0.29 V is recorded for oxo-DPPH [22].

Many other physical or chemical properties of DPPH·, ABTS·^+^ and oxo-DPPH can be compared, and for this purpose, Table 1 shows some such general behaviors.

As seen in Table 1, there are some major differences between these compounds. First of all, only DPPH· is commercially available, while ABTS·^+^ needs to be prepared following a simple procedure (overnight oxidation with persulfate) [23]; in the case of oxo-DPPH, a more elaborate organic synthetic procedure is required [24]. Regarding their solubility, ABTS·^+^ is water-soluble and widely used in TAC measurements in such solvent, as for DPPH· and oxo-DPPH an organic solvent in required (methanol or ethanol is usually used). Although water-methanol mixtures can be used in all cases, this solvent mixture can lead to non-reproducible results, and, additionally, a lack of possibility for comparison with other published data. An interesting fact is that oxo-DPPH showed in our tests slight solubility in water, which was estimated at 10^−5^ M (see the Experimental section); taking into account the high molar extinction coefficient (e = 25,000), this is enough to run such antioxidant measurements using UV–Vis spectroscopy. Although the water solubility seems slight, it is enough for TAC measurements, as oxo-DPPH has the highest e value (Table 1).

The shape of the visible spectrum [25] and the molar extinction coefficient e can be considered the most important properties in TAC measurements (these being related to the UV–Vis spectroscopic band actually used in such measurements).

**Figure 3:**
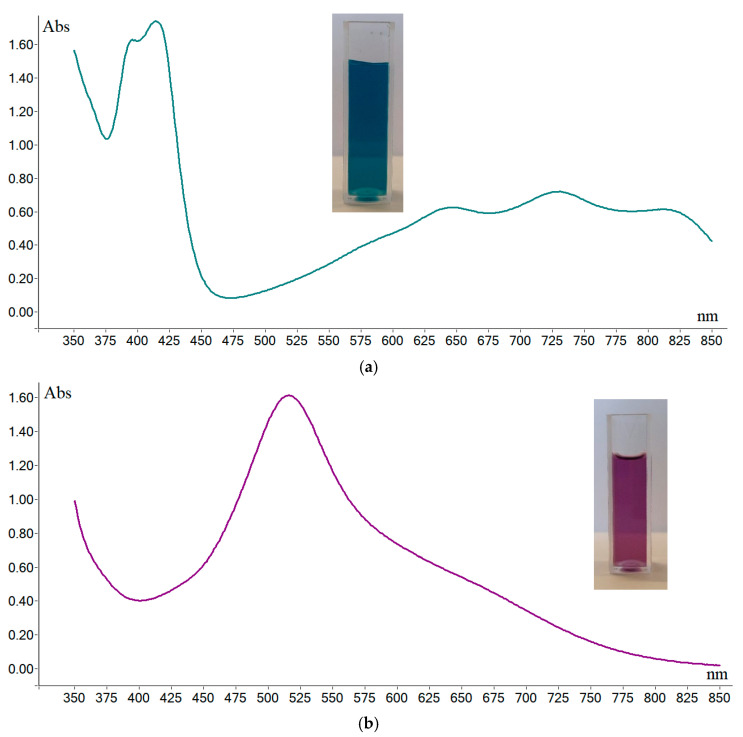

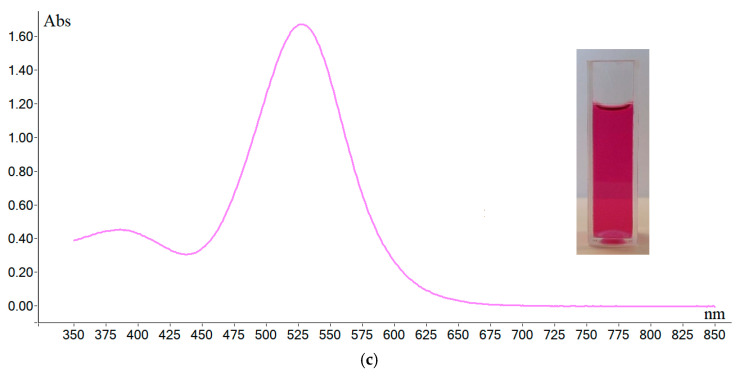
Typical UV–Vis spectra of a solution of (**a**) ABTS·^+^ (in water), (**b**) DPPH· (in methanol), and (**c**) oxo-DPPH (in methanol).

Figure 3 shows the extended visible spectra (including closest UV and NIR part) between 350 and 850 nm, of DPPH·, oxo-DPPH and ABTS·^+^ (and the actual color of the samples).

One advantage of ABTS·^+^ is the possibility to choose over a wide range the correct wavelength to be used in UV–Vis measurements (Figure 3a), which can be an important asset as many samples subjected to TAC measurements are colored. It is therefore necessary to keep possible interferences to a minimum (i.e., if the antioxidant sample absorbs near 400 nm, TAC measurements can be used the values at the other end of the spectrum, like 800 nm, and otherwise). Although DPPH and oxo-DPPH have a single absorption band around 530 nm, oxo-DPPH seems to have after the advantage of sharper band (Figure 3b,c), showing no absorption after about 630 nm, a wavelength at which DPPH still showed high absorption. Therefore, oxo-DPPH might be a better practical working alternative, coupled with the fact that the molar extinction coefficient is practically double compared with that of DPPH. As such, it can definitely be considered a better option.

However, the mechanism of action is another important aspect that was not taken into account in this work [26,27], but several hints are shown in Section 2.4. While DPPH· and ABTS·^+^ are free radicals, oxo-DPPH is a zwitterionic compounds with diradicaloid behavior manifested mainly in solid, and not in solution, as has been demonstrated in previous studies [22,24]. These structural differences can affect the TAC measurements.

### 2.2. Stability Tests of the Solution of DPPH·, ABTS·^+^, and Oxo-DPPH

As free radicals can easily react with substances that contains available electrons and protons (active H-atoms, in fact), a stability study on these compounds was performed, following the changes in absorbance recorded by UV–Vis spectroscopy for the most representative peaks.

**Figure 4:**
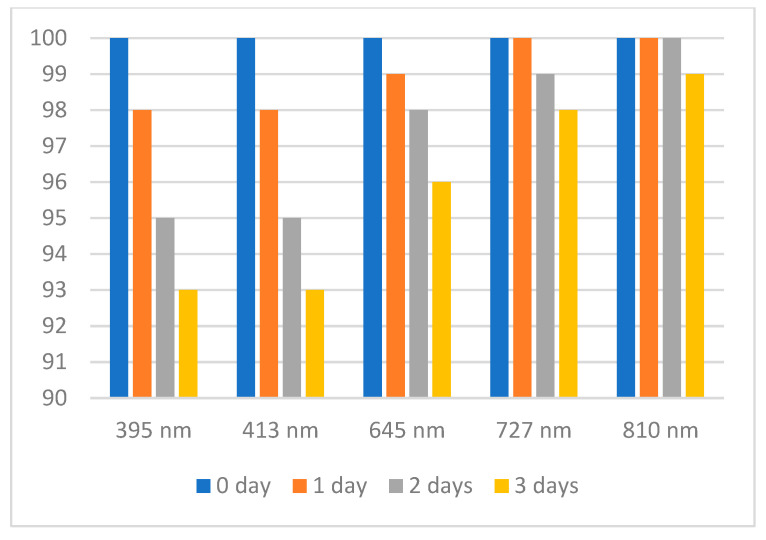
Percentage (%) of decomposition of an aqueous solution for ABTS·^+^ free radical in time (at different wavelengths).

For aqueous ABTS·^+^ free radical solution, the eventual changes in absorbance were monitored for three days, aiming at the peaks recorded at 395, 413, 645, 728, and 811 nm (Figure 1). The results are shown in Figure 4. Thus, it is noticed that after three days, for all the peaks, a decrease in their intensity appears; the highest one was found for the peaks recorded at 395 and 414 nm (7%), and the lowest one for the peak recorded at 811 nm (1%). As a general conclusion, it can be said that it is recommended for TAC measurements to use the peaks that are bathochromic shifted, close to the end of visible domain (~800 nm). In this way, a solution of ABTS·^+^ free radical can be used without major errors even after three days of preparation. As ABTS·^+^ free radical cannot be obtained in organic solvents, no further measurements were made.

**Figure 5:**
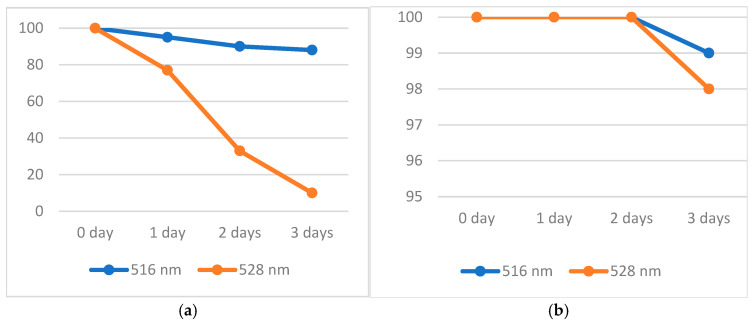
(**a**) Percentage (%) of decomposition of a methanolic solution of DPPH· (blue line) and oxo-DPPH (orange line); (**b**) percentage (%) of decomposition of a DCM solution of DPPH· (blue line) and oxo-DPPH (orange line).

Next, the same test was performed for a methanolic solution of DPPH· and oxo-DPPH, measuring the decrease in absorbance over time at the corresponding peaks (516 and 528 nm, respectively). Figure 5a shows the results obtained. While the solution of DPPH· in methanol slowly fades over time (12% after three days), the oxo-DPPH derivative loses color dramatically (90%). This definitely demonstrates that in all cases fresh solutions of DPPH· and oxo-DPPH are required.

As these compounds are soluble in almost all organic solvents, a supplementary stability test was conducted using dichloromethane (DCM) as the solvent. The results are presented in Figure 5b. A dramatic change was observed, compared with methanolic solutions, showing that both DPPH· and oxo-DPPH are quite stable in DCM for two days, and only on the third day was a small decrease in the absorbance recorded for the corresponding peaks (1 and 2%, respectively). These data concludes that DCM is a better solvent for TAC measurements involving DPPH· and oxo-DPPH.

### 2.3. TAC Measurements

After all these considerations, the next step was the actual use of the proposed oxo-DPPH derivative in practical TAC measurements, as well as a comparison of the results obtained with those obtained from the traditional DPPH· and ABTS·^+^ assays. Thus, as antioxidant was used standard pure ascorbic acid, while as a natural mixture of antioxidants were used extracts of lavender [28], propolis [29,30], and a terpenes mixture used as adjuvant in the pharmacological treatment of several diseases [31,32].

Usually, all natural products contain a blend of different antioxidant compounds, like polyphenols (such as caffeic acid, gallic acid, rosmarinic acid, resveratrol, quercetin), terpenes (limonene, borneol, camphor, borneol, fenchone, anethole, cineol), vitamins (A, C, E), and so on [31,32,33], with well-known antioxidant properties. However, their TAC values are dependent on the method of extraction, the storage conditions, the properties of the raw materials (plants) used (time and region of harvesting), and so on; furthermore, complex mixtures can have peculiar characteristics, as many side-reactions are encountered [34].

**Figure 6:**
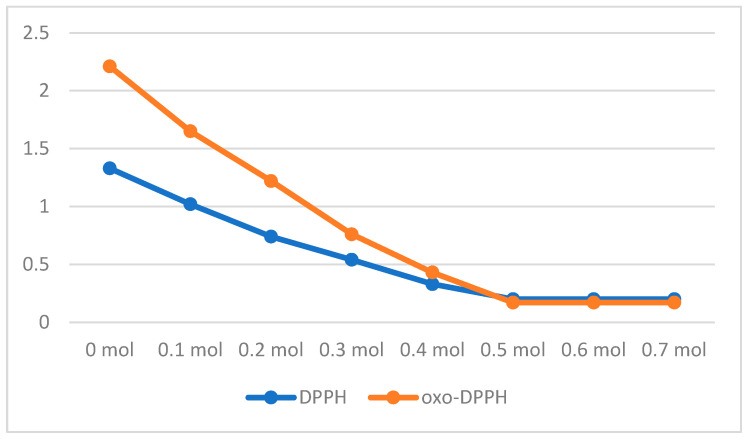
Spectrophotometric titration (*y*-axis Abs as a.u.) of a solution of DPPH· (blue line) and oxo-DPPH (orange line) with ascorbic acid (as equivalent mol).

Starting with ascorbic acid, a colorimetric titration was performed, comparing DPPH with the oxo-DPPH derivative (Figure 6). A freshly prepared methanolic solution of these compounds was thus obtained in methanol, at a concentration of 10^−4^ M. The addition of different molar equivalents of ascorbic acid showed, as expected for DPPH· free radical, color fading at a minimum, after the addition of 0.5 mol equivalents of ascorbic acid (as 1 mol of ascorbic acid reduced 2 mol of DPPH·^+^; see also Table 1). For the case of oxo-DPPH, a surprising result was obtained, showing that color fading required the same amount of ascorbic acid (as mol equivalent). This is completely unexpected, as the reduction in oxo-DPPH to the corresponding HO-DPPH-H counterpart requires two H-atoms (two protons and two electrons (see also Figure 2)).

As for natural antioxidant mixtures (Table 2), the first thing that was noticed was the impossibility of measuring lavender extract with ABTS·^+^. Solutions containing these constituents were inappropriate for UV–Vis measurements, showing a high turbidity and leading to unreproducible results. This is easily explained by the fact that the high oily content of lavender extract make it not soluble in aqueous ABTS·^+^ solution, and thus unsuitable for evaluation. This fault can be corrected using an organic solvent, like with DPPH· and oxo-DPPH.

Thus, Table 2 shows the TAC values obtained for the natural mixtures studied. As mentioned before, the ABTS·^+^ method has the advantage of allowing measurements at different wavelength, but this is limited by aqueous solubility. Regarding DPPH· and oxo-DPPH, TAC values are comparable only for propolis extract; additionally, these are comparable with those obtained following the ABTS·^+^ method. The differences between all three of these methods were somehow expected, and are comparable and compatible with literature data [35].

### 2.4. Mechanism of Action

The widely accepted mechanism for the observed color fading in ABTS·^+^ and DPPH· free radicals solutions is single electron transfer (SET). Thus, in the first stage, both free radicals abstract one electron from the antioxidant substrate, yielding the corresponding anion, followed by an acid–base reaction (proton transfer), that finally lead to the reduced forms. As these information is generally accepted, and being known that oxo-DPPH requires two electrons (and two protons) for reduction, the previously discussed surprising result (0.5 mol equivalents of ascorbic acid is enough to annihilate the color of the oxo-DPPH derivative) requires a more detailed understanding.

**Figure 7:**
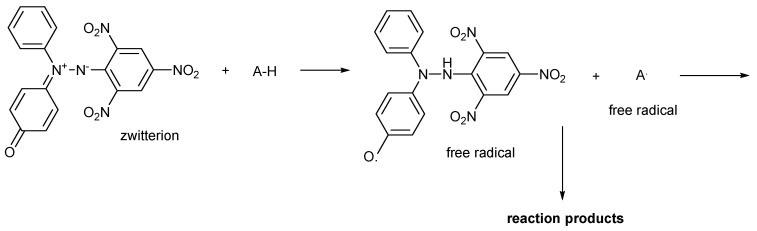
Possible mechanism of action of oxo-DPPH as a non-radical compound used in TAC measurements.

Considering A-H as an antioxidant (like polyphenols or vitamin C), in reaction with oxo-DPPH (Figure 7), is understandable that in fact one H-atom (meaning one-electron and one proton) is necessary to quench the extended conjugation present in the oxo-DPPH molecule. However, from this reaction, two short-lived free radicals are formed, and these can reach a stable form through various ways, like dimerization or a radical + radical reaction. Following this path, it is obvious that in the case of ascorbic acid, although it is a molecule that loose fast two hydrogen atom as reductant, actually it is enough only one to decolorize the oxo-DPPH derivative. No other further experiments were made to support this case; however, the literature [34] is supportive about the possible reactions between the transient radical formed from hydrazyl moiety and other species, including DPPH, showing that this reaction is usually underestimated and can have a contribution regarding the variability of the results.

Some literature data concluded that different reaction mechanisms can be attributed to different assays, including Folin–Ciocalteu (FC), DPPH·, ABTS·^+^, and ORAC [36]; a statistical approach for DPPH·, ABTS·^+^, FRAP, and FC assays is also available [37]. In addition, recommendations were formulated regarding the reaction conditions that need to be adapted for individual groups of antioxidants and an in-depth review of different developments [38,39]. Colored antioxidants can present supplementary issues in terms of their correct evaluation [40].

## 3. Materials and Methods

All chemicals and materials were purchased from Chimopar (Bucharest, Romania) and Merck (Darmstadt, Germany) and used as received. Oxo-DPPH was synthesized according to the literature data [22,24]. Double distilled water was used for ABTS·^+^ solution, while for DPPH· and oxo-DPPH solutions analytical grade methanol and DCM were used. The concentration of ABTS·^+^, DPPH· and oxo-DPPH used for stability and TAC measurements were in the domain of 1–10 × 10^−5^ M.

The spectrophotometric titrations were performed in methanol solution, following the absorbance at 516 nm for DPPH· or 528 nm for oxo-DPPH. The general procedure was as follows: to 1 mL of DPPH· or oxo-DPPH (2 × 10^−4^ M), we added in 0.1 mL of ascorbic acid (2 × 10^−4^ M) in increments (0.0–0.7 mL) and the mixture filled to 2 mL final solution (in this way, in the final solution, the concentration of DPPH· or oxo-DPPH would be 1 × 10^−4^ M. After stirring, the absorbance was measured at the corresponding wavelength.

For all measurements, a double beam UV–Vis spectrophotometer UVD-3500 (Los Angeles, CA, USA) was used. To check reproducibility, most samples were run twice with almost no differences in the measured values. Appropriate solutions of ABTS·^+^, DPPH· or oxo-DPPH (1.9 mL) were mixed with 0.1 mL of antioxidant compounds and left in the dark for 30 min, prior to UV–Vis measurements. The *TAC* values were obtained using the following equation:

$$TAC\;\left(\%\right)=\frac{Abs0-Abs30}{Abs0}\times 100$$

where *Abs*0 is the absorbance value measured for the corresponding peak at time 0 (initially) and *Abs*30 is the value of absorbance measured after 30 min.

The solubility of oxo-DPPH in water was estimated after leaving a mixture of 10 mg of solid oxo-DPPH suspended in 10 mL of double distilled water overnight and under stirring at room temperature. After filtration, the concentration of the pink water solution was estimated by UV–Vis.

## 4. Conclusions

The diversity of the TAC methods is a great asset for such measurements, and their standardization, with the aim of precisely measuring the antioxidant activity, is still an ongoing process. Due to the large composition variety in the structure of the natural compounds, a lot of different factors can influence the practical results, and the search for more specific assays that provide useful information is always upright research. This study aimed to address certain existing limitation of the current assays by proposing the application of the oxo-DPPH derivative. Nevertheless, oxo-DPPH doubtlessly provides several advantages compared with DPPH· free radical: a higher molar extinction coefficient (25,000 versus 11,000); better water solubility (10^−5^ M versus 0); a bathochromic shift in the wavelength maximum (528 nm versus 516 nm), and a sharper shaped visible specific absorption bands. However, oxo-DPPH possesses its own constraints (like slightly more difficult synthesis compared with DPPH·), and further experiments are necessary to validate it as a substitute for DPPH· and ABTS·^+^ free radical in current TAC measurements.

## Figures and Tables

**Table 1:** Comparison of some physical and chemical properties of DPPH·, ABTS·^+^, and oxo-DPPH.

Property	ABTS·^+^	DPPH·	oxo-DPPH
Availability	Simple preparation from ABTS	Commercially available	Single step synthesis from DPPH-H or DPPH·
Solubility	water	organic solvents	organic solvents, water (slightly)
Life-time in solution	weeks	weeks	days-weeks
Life-time in solid	-	stable	stable
Wavelength absorbance (nm) and molar extinction coefficient (e)	395 (35,000) ^ 414 (36,000) 645 (13,000) 728 (16,000) 811 (13,000)	516 (11,000) ^^	385 (6000) ^^ 528 (25,000)
E _ox_	0.50 V	0.34 V	0.29 V
Reduced counterpart	ABTS	DPPH-H	HO-DPPH-H
No. of e^−^ involved	1	1	2
No. of H^+^ involved	1	1	2
Theoretical equivalent ascorbic acid (mol)	1/2	1/2	1
Theoretical equivalent Trolox (mol)	1	1	2

^ in water; ^^ in methanol.

**Table 2:** TAC (%) values obtained using three different assays.

Natural Antioxidants	ABTS·^+^ (Various Wavelengths)	DPPH· (516 nm)	oxo-DPPH (528 nm)
Lavender extract	-	18.64	27.48
Propolis extract	27.77 (811 nm) 30.03 (727 nm) 30.93 (645 nm) 36.82 (413 nm) 37.25 (395 nm)	30.55	27.55
Terpenes mixture	18.80 (811 nm) 20.42 (727 nm) 21.60 (645 nm) 26.07 (413 nm) 28.08 (395 nm)	23.31	15.21

## Data Availability

The original contributions presented in this study are included in the article. Further inquiries can be directed to the corresponding author.
